# Namib Desert primary productivity is driven by cryptic microbial community N-fixation

**DOI:** 10.1038/s41598-018-25078-4

**Published:** 2018-05-02

**Authors:** Jean-Baptiste Ramond, Stephan Woodborne, Grant Hall, Mary Seely, Don A. Cowan

**Affiliations:** 1 Department of Biochemistry, Genetics and Microbiology, Centre for Microbial Ecology and Genomics (CMEG), Genomics Research Institute (GRI), University of Pretoria, Pretoria, South Africa; 2 Mammal Research Institute, University of Pretoria, Pretoria, South Africa; 3 iThemba Laboratories, Johannesburg, South Africa; 4 Gobabeb Research and Training Centre, Walvis Bay, Namibia; 5 Desert Research Foundation of Namibia, Windhoek, Namibia

## Abstract

Carbon exchange in drylands is typically low, but during significant rainfall events (wet anomalies) drylands act as a C sink. During these anomalies the limitation on C uptake switches from water to nitrogen. In the Namib Desert of southern Africa, the N inventory in soil organic matter available for mineralisation is insufficient to support the observed increase in primary productivity. The C4 grasses that flourish after rainfall events are not capable of N fixation, and so there is no clear mechanism for adequate N fixation in dryland ecosystems to support rapid C uptake. Here we demonstrate that N fixation by photoautotrophic hypolithic communities forms the basis for the N budget for plant productivity events in the Namib Desert. Stable N isotope (*δ*^15^N) values of Namib Desert hypolithic biomass, and surface and subsurface soils were measured over 3 years across dune and gravel plain biotopes. Hypoliths showed significantly higher biomass and lower *δ*^15^N values than soil organic matter. The *δ*^15^N values of hypoliths approach the theoretical values for nitrogen fixation. Our results are strongly indicative that hypolithic communities are the foundation of productivity after rain events in the Namib Desert and are likely to play similar roles in other arid environments.

## Introduction

Desert ecosystems cover a substantial portion of the Earth terrestrial surface^1^ and are characterized by very low productivity which is limited by water and nitrogen (N) availability^2–4^. During ubiquitous dry periods, deposited atmospheric or litter-bound N accumulates in these ecosystems and N-mineralization is stimulated only by spatially and temporally stochastic precipitation events^5^. During wet periods, drylands productivity increases exponentially and the regions act as transient carbon sinks^6^. For example, the 2011 global carbon land sink anomaly is attributed in large part to transient desert greening in Australia^6^. Biomass production in desert soils that are nutrient- (particularly N-) deficient, should be limited even after particularly intense wet events^7,8^. This perception is exacerbated by the fact that denitrification, the main process for N-loss in desert terrestrial ecosystems, is also controlled by water availability^9^. It has been estimated that over 99% of the nitrogen fixed by cyanobacterial-dominated biocrusts in arid ecosystems (~8 kg.ha^−1^.yr^−1^)^10^ is lost through this process^11^. Furthermore, desert N fixation has been estimated to range between 4.8 and 10.8 kg.ha^−1^.yr^−1^ 3, which is similar to plant annual uptake in arid lands (12 kg.ha^−1^.yr^−1^)^12^, and at the global scale, atmospheric N land deposition (in the form of nitrate [NO_3_] or ammonium [NH_4_]) is negligible when compared to atmospheric biological N_2_ fixation [BNF]^13^. Consequently, N seems to be lost through denitrification and neither mineralized nor fixed at a sufficient rate to support the rapid and dense vegetation growth observed after pulsed precipitation events in drylands^6^.

In desert ecosystems, microorganisms are believed to be the key drivers of ecosystem processes and services^14,15^. In particular, cyanobacterial-dominated microbial communities found in cryptic niches (biological soil crusts, endo/hypo/chasmoliths) have the potential to actively participate in desert soil C and N budgets as dominant primary producers^10,14–19^. In Antarctic desert soils, hypolithic N-fixation potential was implicated using acetylene reduction assays (ARA) and by the detection of bacterial *nif*H genes^17^. In the Mojave Desert, phototrophic microorganism-dominated hypolithic community N- and C- fixation was shown using stable isotope ratio of Nitrogen (*δ*^15^N), ARA and photosynthetically active radiation (PAR), respectively^16^. A recent shotgun metagenome study also revealed that Namib Desert hypolithic communities could mediate the full N-cycle (apart from ANAMMOX) and possessed substantial capacity for C-fixation as multiple copies of photosystem I and II cyanobacterial genes were detected^18^. A GeoChip microarray analyses of Antarctic Dry Valley hypoliths also recently confirmed that pathways comprising a complete microbially-mediated N-cycle were detected; i.e., N-fixation, (de)nitrification, ammonification, assimilatory/dissimilatory nitrogen reduction (A/DNR), and ANAMMOX^19^.

In deserts where microbial colonized rocks (hypo-, endo- and/or chasmo-liths) are highly distributed, their cryptic communities may represent a likely nutrient cycling hub providing desert soils with sufficient mineralized N to support plant growth. However, this hypothesis has never been experimentally confirmed in the field. In this study, we have used natural stable isotope ratios of Nitrogen (*δ*^15^N) and Carbon (*δ*^13^C) to evaluate if desert hypoliths constitute atmospheric N fixation hubs^20,21^.

## Results

**Figure 1:**
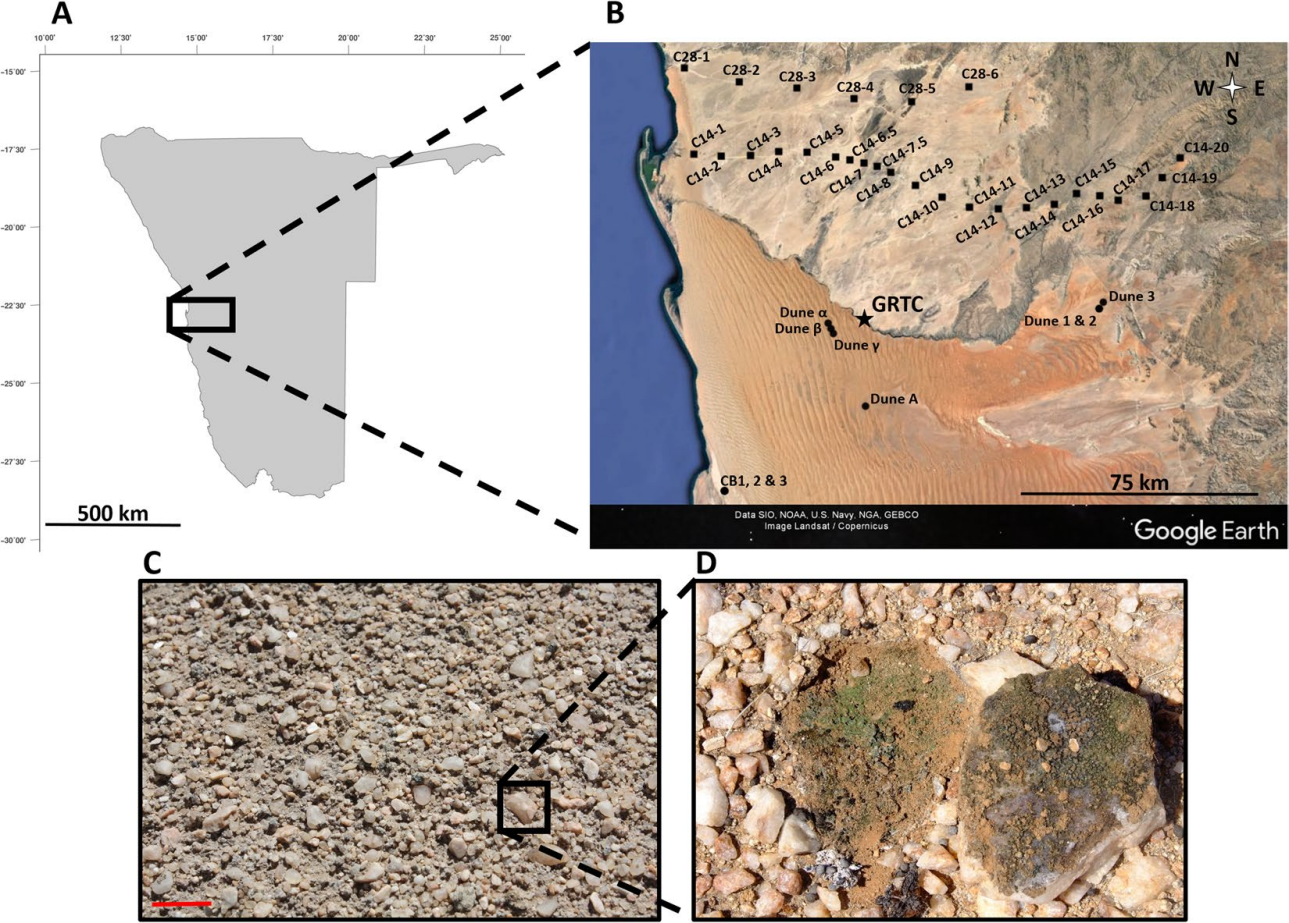
Map of Namibia (**A**) showing the distribution of the sampling sites (**B**) in the Central Namib Desert. ■ Gravel plain. ● Dune. (**C**) Photograph of the Namib Desert gravel plain quartz rock pavement. Scale bar represents 10 cm. (**D**) Close-up picture of an overturned quartz rock. The green biomass the ventral surface of the rock and on the soil just under the overturned rock is indicative of a cyanobacterial-dominated microbial community and primary production. The map of Namibia was adapted from one obtained from the GinkgoMaps-project (http://www.ginkgomaps.com/maps_namibia.html) and the Central Namib Desert map was produced with Google Earth, © 2016 DigitalGlobe. *Photo courtesy of J-B Ramond and DA Cowan*.

Soil and hypolithic samples were collected in the gravel plains and the dune fields of the central Namib Desert over a period of 3 years (Fig. 1; Supplementary Table 1). The full analytical dataset (*δ*^13^C, *δ*^15^N, %C, %N and C/N) of the 152 samples (hypoliths: n = 52; surface soils: n = 53; subsurface soils: n = 47) is provided in Supplementary Table 1. Of the thirteen SASSCAL weather stations spanning the sampling sites at the time of sampling, only two (Ganab once and Dieprivier thrice) recorded precipitation events over 20 mm a month before sampling and the sampling month (Supporting Table 2). This amount has been identified as the minimal necessary for C4 grasses, which cannot fix atmospheric N, to grow in the Namib Desert gravel plains^22^. In March and April 2014, 2015 and 2016 only 3 of the 6, 4 of the 26 and 3 of the 26 monthly cumulative precipitations records, respectively, represented more than 5 mm and precipitation was highly localized both in time and space (Supporting Table 2). Consequently, this strongly indicates that the C and N chemistries measured in this study were globally not influenced by atmospheric N deposition nor plant decay.

**Figure 2:**
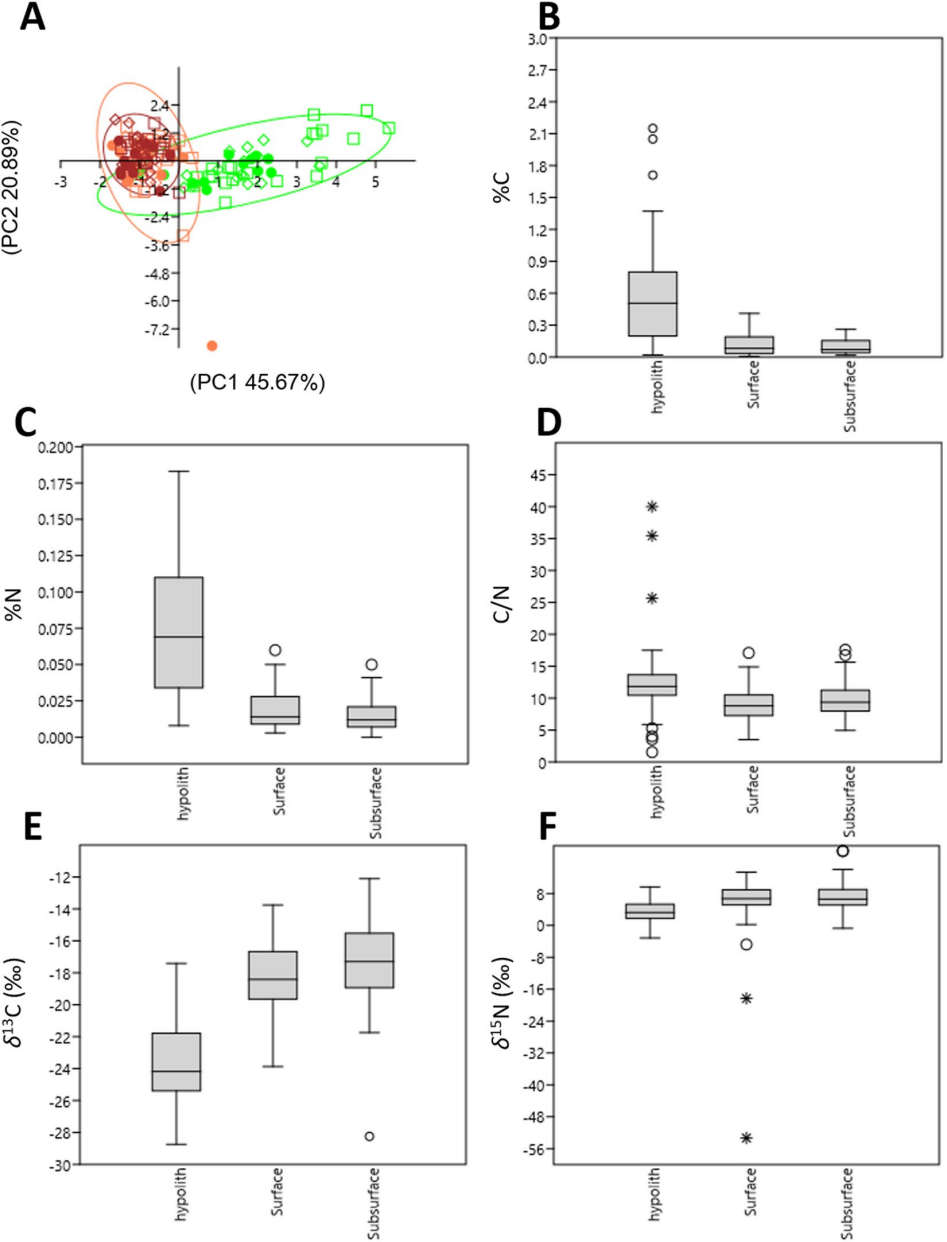
Namib Desert hypolith, surface and subsurface soil C and N chemistry comparative analyses. (**A**) PCA plot of C and N chemistries. Hypoliths are indicated in green, surface soils in orange and subsurface soils in brown. ● 2016; ◊ 2015; □ 2014. Ellipses indicated 95% confidence. (**B**) Habitat-specific boxplot of %C. (**C**) Habitat-specific boxplot of %N. (**D**) Habitat-specific boxplot of C/N ratios. (**E**) Habitat-specific boxplot of *δ*^13^C. (**F**) Habitat-specific boxplot of *δ*^15^N. Boxplot outliers are indicated by ○ and *.

**Figure 3:**
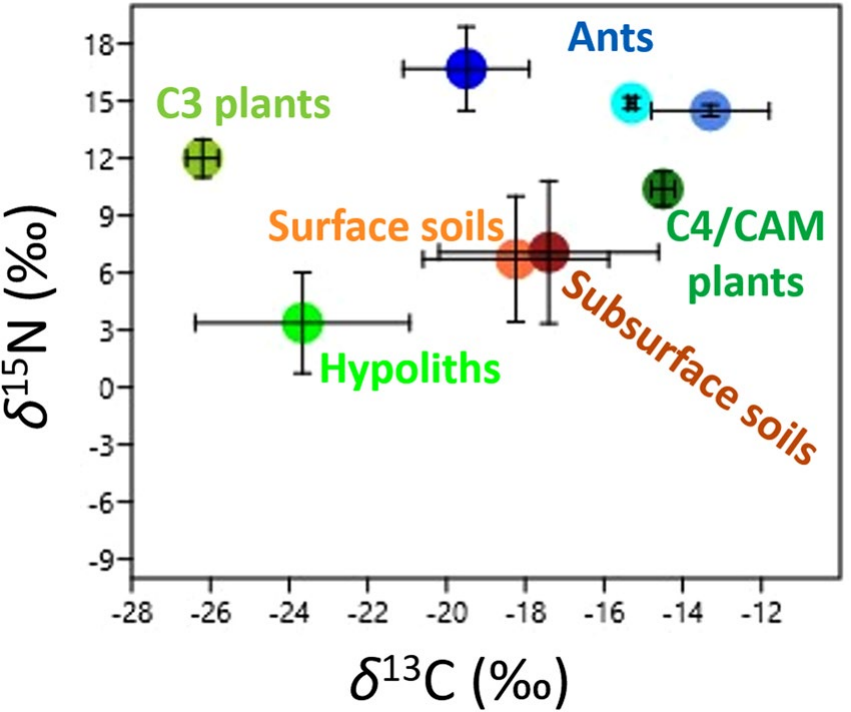
Bi-plot of mean (±SD) stable isotope compositions (*δ*^13^C and *δ*^15^N; ‰) from Namib Desert biosystems. Plants and ants isotopic signatures were obtained from^41^. Outliers identified in Fig. 2 were excluded from the analysis.

The edaphic and hypolithic C and N chemistries were found to be independent of the year of sampling (Kruskal-Wallis H, p > 0.05; Table 1) while they were globally significantly different in the different environments studied (Kruskal-Wallis H and Dunn’s pairwise tests, p > 0.05; Table 1; Fig. 2A). Pairwise comparison showed that hypoliths presented significantly different N and C chemistries from the soils but that surface and subsurface soils did not (Kruskal-Wallis H and Dunn’s pairwise tests, p < 0.05; Table 1, Fig. 2). Hypoliths presented significantly higher %N, %C and C/N ratios and lower *δ*^13^C values than soils (p < 0.05; Table 1; Fig. 2B,C and D) but respectively higher and lower *δ*^13^C values than Namib Desert C3- and C4-plants (Fig. 3). When excluding the two surface soils outliers indicated by “*” in Fig. 2F (δ^15^N = −53.3 [Site C14-5 in 2016] and *δ*^15^N = −18.31 [Site C14-6 in 2014]; Supplementary Table 1), hypoliths also displayed significantly lower *δ*^15^N values (Dunn’s pairwise test, p < 0.05, Table 1, Fig. 2E) than (sub)surface soils and Namib Desert C3 and C4/CAM plants (Fig. 3). Overall, dune and gravel plain *δ*^13^C and *δ*^15^N values were not significantly different (Kruskal-Wallis H, p > 0.05; Table 2) while the %N (0.04 [±0.04] vs 0.02 [±0.03]), %C (0.31 [±0.39] vs 0.10 [±0.16]) and C/N (10.97 [±4.61] vs 7.46 [±2.91]) were significantly higher in the Namib Desert gravel plains than in the Namib Sand Sea (Kruskal-Wallis H, p < 0.05; Table 2, Supplementary Table 1).

**Table 1:** Kruskal-Wallis H test results testing the effect of the year of sampling and of the environment on C and N chemistry with Dunn’s pairwise comparison test results.

		Kruskal-Wallis test		Dunn’s pairwise test		
		H	p	2016/2015	2016/2014	2015/2014
Year of sampling	*δ*^15^N	4.461	0.1075	0.1281	0.2678	1
	*δ*^13^C	0.342	0.8428	1	1	1
	%N	3.684	0.158	1	0.2105	0.597
	%C	3.3358	0.1864	0.9415	0.2024	1
	C/N	4.654	0.09756	0.02409	0.1172	1
		**H**	**p**	**Hypolith vs Surface**	**Hypolith vs Subsurface**	**Surface vs Subsurface**
Environment	*δ*^15^N	36.19	<0.0005*	<0.0005*	<0.0005*	1
	*δ*^13^C	79.82	<0.0005*	<0.0005*	<0.0005*	0.5118
	%N	63.82	<0.0005*	<0.0005*	<0.0005*	1
	%C	60.02	<0.0005*	<0.0005*	<0.0005*	1
	C/N	25.5	<0.0005*	<0.0005*	<0.0005*	0.7315

H: Kruskal-Wallis test H(chi^2^) statistic. *Significantly different (p < 0.05).

**Table 2:** Kruskal-Wallis H test results testing if there was a significant difference in the C and N chemistries of samples from different Namib Desert habitat (dune or Gravel plain) or precipitation regime zones (fog vs rain).

		Kruskal-Wallis test	
		H	p
Dune vs Gravel plain	*δ*^15^N	1.371	0.2417
	*δ*^13^C	0.5169	0.4722
	%N	13.61	<0.0005*
	%C	22.23	<0.0005*
	C/N	19.33	<0.0005*
Fog vs Rain	*δ*^15^N	7.286	<0.01*
	*δ*^13^C	12.35	<0.0005*
	%N	22.34	<0.0005*
	%C	26.49	<0.0005*
	C/N	1.7	0.1923

H: Kruskal-Wallis test H(chi^2^) statistic. *Significantly different (p < 0.05).

**Figure 4:**
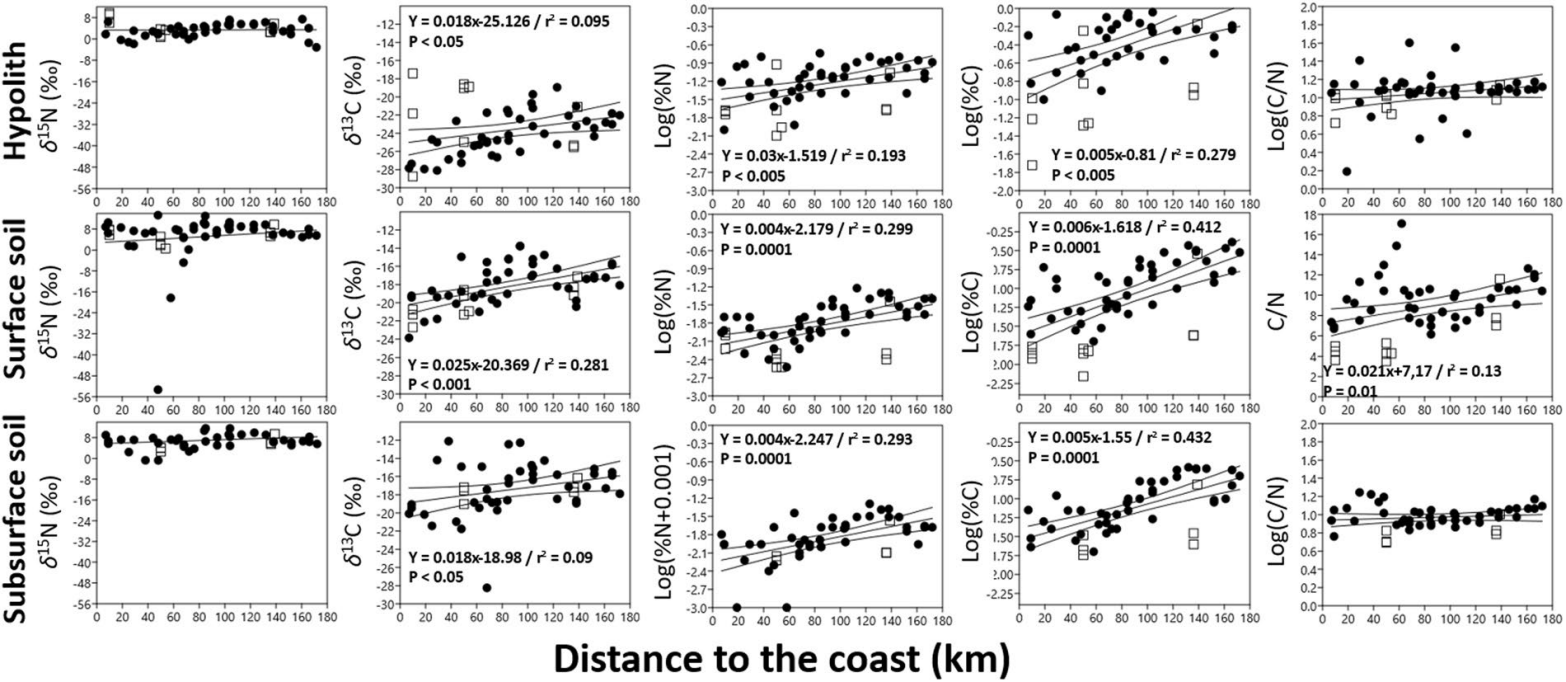
Spatial relationships between Namib Desert hypolithic and edaphic C and N chemistries with the distance to the coast. When significant (p < 0.05) relationships are indicated in the plot along with OLS regression equations and r^2^ values. Bootstrapped 95 percent confidence intervals (1999 replicates) border the regression line. □ Dune; ● Gravel Plain.

The sample set spanned a naturally occurring longitudinal xeric stress gradient with two dominant water sources with coastal fog extending up to 75 km inland (see Supplementary Table 1)^23–25^. The rain-zone samples consistently showed significantly higher *δ*^13^C (−19.1 [±3.64] vs −21.17 [±3.76]), *δ*^15^N (6.23 [±3.63] vs 4.64 [±3.44]), %N (0.04 [±0.04] vs 0.02 [±0.03]) and %C (0.35 [±0.42] vs 0.14 [±0.22]) values (Kruskal-Wallis H, p < 0.05; Table 2, Supplementary Table 1) relative to the fog zone samples. In hypoliths, surface and subsurface soil samples from the rain zones when compared to those of the fog zones the *δ*^13^C (−23.21 [±1.96] vs −24.43 [±3.63], −17.14 [±1.76] vs −20.14 [±1.91] and −16.97 [±2.71] vs −18.41 [±2.77], respectively), %N (0.09 [±0.04] vs 0.05 [±0.04], 0.03 [±0.01] vs 0.009 [±0.006] and 0.02 [±0.01] vs 0.007 [±0.006], respectively) and %C (0.75 [±0.52] vs 0.32 [±0.30], 0.17 [±0.11] vs 0.05 [±0.05] and 0.13 [±0.08] vs 0.04 [±0.03], respectively) values were also significantly higher (Kruskal-Wallis H, p < 0.05; Table 3, Supplementary Table 1). Hypoliths, surface and surface soils from the rain and fog samples from the dunes (Table 3) show the same trends in the gravel plains, with higher *δ*^13^C, %C and %N in rain-zones (Kruskal-Wallis H, p < 0.05; Table 3, Supplementary Table 1). A significant positive linear relationship between the ‘distance to coast’, which is a proxy for MAP in the central Namib Desert (see Supplementary Table 1)^23,24^, and the %N, %C and *δ*^13^C values of the hypoliths, surface and subsurface soils was also identified (Fig. 4). This further demonstrated that water availability is a crucial determinant in desert C and N cycling.

**Table 3:** Kruskal-Wallis H test results testing the effect sample moisture source (fog vs rain) on the C and N chemistries of samples from the Namib Desert environments tested globally or when originating from different habitats (dune vs gravel plain).

		*δ*^15^N		*δ*^13^C		%N		%C		C/N	
		H	p	H	p	H	p	H	p	H	p
Global test	Hypolith	2.64	0.1042	3.943	0.0471*	8.509	0.003*	12.63	<0.0005*	2.113	0.146
	Surface soil	2.88	0.08	18.84	<0.0005*	17.25	<0.0005*	19.91	<0.0005*	4.432	0.035*
	Subsurface soil	4.325	0.038*	6.318	0.012*	14.13	0.0002*	15.67	<0.0005*	0.001	0.9734
Dune	Hypolith	0.3247	0.5688	1.571	0.21	2.195	0.1337	2.195	0.1385	2.195	0.1385
	Surface soil	1.571	0.21	4.688	0.03*	0.3247	0.5653	5.727	0.016*	5.727	0.0167*
	Subsurface soil	3.857	0.049*	0.4286	0.5127	3.857	0.0431*	2.333	0.1266	2.333	0.1266
Gravel plain	Hypolith	6.419	0.0112*	15.41	<0.0005*	4.36	0.0366*	6.633	0.01*	0.063	0.8
	Surface soil	0.4956	0.4814	10.04	0.002*	11	0.0009*	11.55	0.0007*	0.005	0.946
	Subsurface soil	1.712	0.1907	5.149	0.02325*	10.43	0.0012*	12.9	<0.0005*	1.005	0.3162

H: Kruskal-Wallis test H(chi^2^) statistic. *Significantly different (p < 0.05).

## Discussion

Hypolithons are microbial communities colonizing the ventral surfaces of translucent rocks, mainly quartz, and are commonly found in hot and cold desert pavement environments (Fig. 1B,C)^1,26,27^. The lithic substrate provides the underlying communities with a stable substrate with sufficient transmitted incident light to support ‘cryptic photosynthesis’, while protecting them from extreme environmental conditions (e.g. UV radiation and desiccation)^27,28^ and buffering (rapid) changes in microenvironmental parameters^27^. In the gravel plains of the Namib Desert, hypolithic communities have been shown to selectively recruit their constituent microbial species from the surrounding soils, to be dominated by primary producers, i.e., cyanobacteria (notably *Chroococcidiopsis* sp.), and to support a wide range of heterotrophic taxa^29,30^. Cyanobacteria, and particularly a cryptic cyanobacterial Operational Taxonomic Unit (OTU) assigned to the genus GpI, were also found to drive hypolithic food webs based on co-occurrence network analyses^30^.

Our chemical analyses show that Namib Desert hypolithic biomass and soils are highly oligotrophic habitats. However, hypoliths were significantly less nutrient-limited than desert soils (Fig. 2B,C; Table 1), which is consistent with previous genetic evidence for high C- and N-fixing capacities^18,19^. Nutrient stratification in surface and subsurface soils was not observed (Fig. 2B,C; Table 1)^14^ which confirmed that nutrient cycling is very limited in Namib Desert soils^31^. Edaphic and hypolithic C and N chemistries were also found to be independent of the year of sampling (Table 1), most probably reflecting their local and long-term “hydro-histories”^32,33^. Moisture source (i.e., fog vs rain) has previously been found to impact hypolithic community structures^30^ in the central Namib Desert. Similarly, hypolithic colonization has been shown to be highly correlated to water availability in the central Namib Desert^34^ and at the global scale^27^. Edaphic bacterial community structures and functions have also recently been found to be influenced by moisture source^35^. This is consistent with our finding that %N and %C values were significantly higher in the rainfall zone (Fig. 4, Table 2, Supplementary Table 1) and that a linear and statistically significant increase in %C and %N along a west-east longitudinal transect was observed (Fig. 4). Since the mean annual precipitation (MAP) is higher in the rain zone than in the coastal fog zone and increases from the coast inland^23,24^, the significantly higher biomass observed in the rain zone samples (%N and %C, p < 0.005, Tables 2 and 3, Supplementary Table 1) almost certainly reflected increased edaphic and hypolithic microbial activities^35,36^.

Both Namib Desert soils and hypoliths have *δ*^13^C and *δ*^15^N values typical of arid environments^16,33,37,38^; both being significantly lower in the hypoliths (Fig. 3). With averaged *δ*^13^C values of −23.66 (±2.72) ‰ (Fig. 2E), Namib Desert hypoliths show a photosynthetic signature typical of C3-plants^39,40^. Surface and subsurface soils presented δ^13^C signatures located between those of the Namib Desert C3 plants/hypoliths and C4 plants (Fig. 3), indicating most probably that Namib Desert soils’ C originate both by hypolithic and C3/C4 plant fixation and decaying of plant material. *δ*^15^N values close to 0‰ are characteristic of N acquisition via microbial fixation, values between +2 to +5‰ are typical of atmospheric N-fixation by fungal mycorrhizae or a mix of N-fixation and mineralized N from soils, and values >6‰ are the result of uptake of mineralized N^20,37^. Averaged *δ*^15^N values for Namib Desert hypolith samples were 3.38 (±2.65) ‰ (Fig. 2F), which suggests that they derive their N via microbial (bacterial and/or fungal) fixation of atmospheric-N (as previously observed for Mojave Desert hypoliths which presented *δ*^15^N values of 0.6 (±0.18) ‰^16^), subsequently mineralized by ammonifying bacteria. This is supported by the detection of Cyanobacterial and Proteobacterial *nif*H sequences and contigs from *Nitrosomonas* sp., *Nitrobacter* sp. and *Nitrospira* sp. in a Namib Desert hypolith metagenome^18^.

δ^15^N values for hypoliths were significantly lower than those of Namib Desert soils and plants (Fig. 3)^41^. Since discrimination of *δ*^15^N evolves positively in a system^42^, these results further demonstrate that (hot) deserts hypolithic communities are a N-fixation hub that positively contribute to N-fertilization of dryland soils. This result is fundamental in better understanding desert ecosystem functioning. While desert plants acquire C for growth through autotrophic photosynthetic activity, they require bioavailable N from soils for both growth and/or increases in photosynthetic capacity^43^.

Desert soils are indeed globally N-limited^7,8^, but during wet anomalies, desert macrophytic plants (mainly C4 grasses) that cannot fix atmospheric N manage to acquire sufficient N to support rapid and substantial growth^6,43^. This has also been observed in the N-depleted gravel plain soils of the Namib Desert where the C4 short-lived perennial grass S*tipagrostis ciliata* grows extremely fast and covers their surface after precipitation events over 20 millimetres^22,44^. These plains are largely devoid of N-fixing plants such as *Vachellia* spp. that might increase the edaphic N supply. Our results therefore suggest that the bioavailable N necessary for *S. ciliata* to demonstrate such rapid growth in the Namib Desert after a rain event in the Namib Desert gravel plains is largely provided by the N-fixing capacity of hypolithic microbial communities. On the basis of *δ*^15^N values, it was suggested that plants acquire bioavailable N in the Atacama Desert from intermediate N-fixing hubs, such as lichens^45^ and/or hypoliths^38^. They demonstrated that the plants did not directly obtain their N from fog precipitation; even in zones where fog constitutes the sole water source.

## Conclusions

In unmanaged terrestrial systems, Biological Nitrogen Fixation is the primary process by which N enters the system^3,13,46^. BNF has also been found to be regulated by climate and positively correlated with moisture availability^11,47^. Consequently, we argue that hypolithic microbial communities, which are positioned at the bottom of the Namib Desert primary production web (Fig. 3), fix N_2_ and produce sufficient bioavailable N (in the form of ammonium or nitrate^20^) during wet anomalies to support their own growth as well as the growth of higher plants. The fact that 98% of the quartz rocks over 5 cm were colonized in a similar transect in the Namib Desert gravel plains, and that this coverage is independent from moisture source (fog vs rain, which conversely significantly impacted the Namib Desert hypolithic C and N chemistries; Fig. 4, Tables 2 and 3, Supplementary Table 1), strongly supports this view^34^. We therefore suggest that when hypoliths are abundant, as in the Namib Desert gravel plains^34^, they are a critical foundation of hot desert productivity via their capacity for N-fixation. *In situ* monitoring of *S. ciliata* growth in the Namib Desert (e.g., number of plants per m^2^) in relation with the abundance of colonized hypoliths after controlled or natural 20 mm rain events in parallel with stable isotopic tracer studies^48^ sh/could be performed to further demonstrate this point. Our results also particularly indicate that depending on the percent colonization and the distribution of colonized stones, desert plant response to rain could vary. For example, in deserts where quartz stone colonization vary, such as in the Atacama (from 27.6% to 0% in the semiarid and hyperarid regions respectively)^49^ or the Taklimakan and Qaidam Basing deserts (from 12.6% to 0% along an aridity gradient)^50^, plant growth should be patchy after an intense rain event, while in deserts presenting ‘constant’ hypolithic colonization, such as the Namib Desert (~98% in every undisturbed gravel plain sites visited along the same aridity and fog/rain gradient we studied here)^34^, plant growth should be uniform.

The process rates of hypoliths, and also other cyanobacterial-dominated cryptic microbial communities (e.g., endolithic communities^14^), during dry spells and wet anomalies, as well as plant growth after substantial rain events, should be characterized quantitatively in different deserts worldwide in order to be included in future climate models^51,52^. This is fundamental as hypoliths can cover up to 50% of dryland surfaces^1^, and thus arid lands may contribute more to global C- and N-cycling than previously estimated^10,53,54^.

Finally, Warren-Rhodes and colleagues^34^ have observed that the colonization of quartz stones in the Namib Desert gravel plains were significantly lower in disturbed (i.e., only from 50% to 70% in highly disturbed and moderately disturbed sites, respectively) than in undisturbed (~98%) sites. This suggests that hypolithic communities need long-term soil stability to develop and shows that they are susceptible to environmental changes. Our study, therefore, further indicates that ecological restoration in the Namib Desert gravel plains, after mining for example^55^, may strongly depend on translucent rock re-colonization and thus be a rather long process.

## Methods

### Sample collection

Namib Desert hypoliths (n = 52) and surface (0–2 cm; n = 53) and subsurface (15–20 cm; n = 47) soil samples were collected aseptically in the dunes and gravel plains of the central Namib Desert in April 2014, 2015 and 2016 (Fig. 1; Supplementary Table 1). At each sampling site, hypolithic biomass was scraped off the undersides of 3 to 5 translucent rocks collected within a 5 m radius, and pooled. At each site, this has led to the recovery of ~5 g of hypolithic biomass. Surface soil samples were collected from a hypolith-free area within the same 5 m radius and the subsurface soil samples immediately below them. All samples were kept at room temperature in sterile 15 mL falcon tubes prior to their analysis at the Stable Isotope Laboratory of the Mammal Research Institute, University of Pretoria.

### Climatic data

The Namib Desert is characterized by an east/west longitudinal rainfall gradient which increases from the coast inland and by regular coastal fog events which can penetrate ~70 km inland^23,24^. The dominant moisture source (fog *vs* rain; see Supplementary Table 1) of each sample was defined based on extensive climatological data records^23,24^. The CRUTS v.3.24 high resolution climate data set (https://crudata.uea.ac.uk/cru/data/hrg/)^25^, spanning 1901–2015, was used to determine Mean Annual Precipitation (MAP, mm) in half degree grids (25 km^2^) of the Namib Desert (see Supplement for the MAP values reported for each sampling site).

We also provide monthly precipitation records of 13 central Namib Desert weather stations from the Southern African Science Service Centre for Climate Change and Adaptive Land Management (SASSCAL) network (http://www.sasscalweathernet.org/)^56^ for March and April 2014, 2015 and 2016, i.e. the month prior to sampling and the sampling month (Supplementary Table 2). The SASSCAL weather stations were chosen based on their proximity to the sampling sites. Twelve SASSCAL weather stations are located in the central Namib Desert gravel plains and one far west in the central Namib Desert dune fields.

### C and N analyses

Untreated aliquots were used for Nitrogen isotope measurements, while aliquots treated with a 1% HCl (v/v) solution (to remove all inorganic carbonates) were used for Carbon isotope value measurement. The samples were repeatedly washed with distilled water to neutral pH and dried at 70 °C. Aliquots of approximately 80 to 100 mg were weighed into tin capsules pre-cleaned in toluene and analysed using a Flash EA 1112 Series elemental analyzer coupled to a Delta V Plus stable light isotope ratio mass spectrometer via a ConFlo IV system (all equipment from Thermo Fischer, Bremen, Germany).

A laboratory running standard (Merck Gel: *δ*^13^C = −20.57‰, *δ*^15^N = 6.8‰, C% = 43.83, N% = 14.64) and a blank sample were run after every 5 unknown samples. All results were referenced to Vienna Pee-Dee Belemnite for carbon isotope values and to air for nitrogen isotope values. Results are expressed in delta (*δ*) notation using a per mille scale using the standard equation:

$$\delta {\rm{X}}({\mathbb{\textperthousand }})=[({\rm{R}}\,{\rm{sample}}-{\rm{R}}\,{\rm{standard}})/{\rm{R}}\,{\rm{standard}}-1]\times 1000$$

where X represents ^15^N or ^13^C and R represents the ^15^N/^14^N or the ^13^C/^12^C ratio, respectively. Analytical precision was <0.09‰ for *δ*^13^C and <0.08‰ for *δ*^15^N.

### Statistical analyses

Statistical analyses were performed using the PAST v3.14 software package. Principal component analysis (PCA) was performed on normalised datasets and based on Euclidean distances. Kruskal-Wallis H tests with pairwise Dunn’s post hoc test were used to identify significant differences between samples from different environments (hypoliths *vs* surface soils *vs* subsurface soils), origin (Dune vs Gravel plain), years of sampling (2014 vs 2015 vs 2016) or moisture sources (fog *vs* rain) (Tables 1, 2 and 3). A total of 9999 random permutations were performed and p values were Bonferroni-corrected. Ordinary Least Square (OLS) was used to evaluate linear relationships between ‘distance-to-coast‘ and hypolithic and edaphic *δ*^13^C, *δ*^15^N, %N, %C or C/N ratios. Hypolithic %N, %C and C/N ratios and subsurface %N were log-transformed to achieve near-normal distribution. Soil surface *δ*^13^C, *δ*^15^N, C/N, log(%N) and log(%C) and subsurface %C, and C/N were normally distributed.

## Electronic supplementary material

Supplementary information
