# Supplementary material for: Namib Desert primary productivity is driven by cryptic microbial community N-fixation
Source: Sci Rep. 2018 May 2;8:6921. doi: 10.1038/s41598-018-25078-4 (PMC5932006; doi:10.1038/s41598-018-25078-4)
Supplement: Supplementary file 1 — Supplementary information [file 41598_2018_25078_MOESM1_ESM.pdf]

Supplementary Information

Namib Desert primary productivity is driven by cryptic microbial community N-fixation

Jean-Baptiste Ramond[1,*], Stephan Woodborne[2,3], Grant Hall[2], Mary Seely[4,5], Don A Cowan[1]

[1] Centre for Microbial Ecology and Genomics (CMEG), Genomics Research Institute (GRI), Department of Genetics, University of Pretoria, South Africa

[2] Mammal Research Institute, University of Pretoria, South Africa

[3] iThemba Laboratories, South Africa

[4] Gobabeb Research and Training Centre, Walvis Bay, Namibia.

[5] Desert Research Foundation of Namibia, Windhoek, Namibia

Supplementary Table 1. Metadata of the sample set studied. The dominant moisture source (fog vs rain, Supplementary Table 1) of each sample was based on extensive climatological data records (Lancaster et al., 1984; Eckardt et al., 2013). The CRU TS v. 3.24 high resolution climate data set, spanning 1901-2015, was used to determine Mean Annual Precipitation (MAP, mm) in half degree grids (25 km2) of the Namib Desert (Supplementary Table 1; Harris et al, 2014; https://crudata.uea.ac.uk/cru/data/hrg/).

Environment	Year of sampling	Origin	Moisture Source	Sample Site Name	Distance to the coast (km)	Altitude (m)	Mean Annual Precipitation (mm)	δ 15N (‰)	δ 13C (‰)	%N	%C	C/N
**Hypolith (n=52)**	2016	Gravel Plain	Fog	C14-3	29	233	0,46	3.05	-24.99	0.035	0.198	8.88
				C14-5	48	415	0,46	2.15	-27.26	0.024	0.193	12.22
			Rain	C14-7	68	570	2,41	2.2	-24.88	0.034	0.257	11.45
				C14-9	85	686	2,41	3.9	-24.18	0.086	0.795	13.28
				C14-11	104	830	2,41	5.05	-23.22	0.103	0.552	12.76
				C14-13	123	932	6,54	5.31	-25.22	0.068	0.573	12.2
				C14-15	138	880	6,54	4.77	-21.03	0.073	0.589	11.69
				C14-17	152	1035	6,54	1.77	-24.34	0.105	0.486	11.54
				C14-19	166	1083	15,47	4.03	-23.01	0.086	0.65	12.38
		Dune	Fog	Dune a	50	392	0,13	1.2	-24.96	0.119	0.568	10.42
				Dune ß	50	406	0,13	0.67	-18.62	0.021	0.15	10.91
				Dune γ	50	406	0,13	3.67	-19.03	0.008	0.052	7.73
	2015	Gravel Plain	Fog	C14-6.5	62	573	2,41	1.75	-25.26	0.043	0.576	14.78
			Rain	C14-7	68	570	2,41	1.6	-25.04	0.069	0.474	10.8
				C14-7.5	72	579	2,41	-0.15	-26.44	0.083	0.587	11.09
				C14-8	76	615	2,41	1	-24.76	0.052	0.67	12.21
				C14-9	85	686	2,41	2.43	-21.79	0.077	0.885	12.71
				C14-10	94	751	2,41	3.24	-22.43	0.076	0.69	11.34
				C14-11	104	830	2,41	7.13	-21.23	0.108	0.91	10.44
			Fog	C28-1	7	55	1,83	1.77	-27.83	0.061	0.506	11.29
				C28-2	25	211	1,83	-1.24	-24.68	0.121	1.126	13.97
				C28-3	44	355	1,83	2.81	-22.65	0.061	0.374	11.84
		Dune	Rain	C28-4	64	467	2,41	4.68	-24.46	0.012	0.125	14.37
				C28-5	84	704	2,41	4.74	-21.45	0.183	0.812	11.35
				C28-6	103	938	11,28	5.41	-20.69	0.075	0.621	11.54
				Dune 1	136	928	4,75	2.62	-25.34	0.022	0.112	9.54
				Dune 2	136	928	4,75	2.47	-25.53	0.021	0.135	12.08
				Dune 3	139	935	4,75	5.7	-21.09	0.087	0.675	13.74
	2014	Gravel Plain	Fog	C14-1	9	22	1,83	6.4	-27.37	0.01	0.15	14.11
				C14-2	19	118	1,83	-0.37	-27.94	0.11	0.1	1.55
				C14-3	29	233	0,46	-1.88	-28.1	0.06	0.86	25.66
				C14-4	38	312	0,46	1.23	-26.88	0.16	0.35	6.15
				C14-5	48	415	0,46	1.72	-26.3	0.04	0.27	15.09
				C14-6	58	535	2,41	3.86	-25.39	0.03	0.31	12.97
			Rain	C14-7	68	570	2,41	2.92	-21.73	0.05	0.8	40.01
				C14-8	76	615	2,41	4.19	-26.65	0.11	0.3	3.54
				C14-9	85	686	2,41	4.3	-24.8	0.04	0.36	17.49
				C14-10	94	751	2,41	5.34	-26.04	0.07	0.3	5.88
				C14-11	104	830	2,41	6.49	-19.71	0.04	1.27	35.44
				C14-12	113	936	6,54	5.54	-24.05	0.13	0.27	4.04
				C14-13	123	932	6,54	5.7	-18.94	0.16	2.05	13.66
				C14-14	132	881	6,54	6.24	-22.07	0.13	1.37	11.47
				C14-15	138	880	6,54	2.81	-23.22	0.14	1.71	13.04
				C14-16	146	960	6,54	2.84	-22.66	0.16	2.15	12.53
				C14-17	152	1035	6,54	3.88	-23.43	0.04	0.32	14.56
				C14-18	161	1085	15,47	7.39	-22.82	0.14	1.36	12.32
				C14-19	166	1083	15,47	-1.47	-21.81	0.07	0.59	14.94
				C14-20	172	1255	15,47	-3.14	-22.02	0.13	1.25	13.2
		Dune	Fog	CB1	10	9	0,13	9.04	-21.83	0.018	0.019	5.28
				CB2	10	10	0,13	6	-28.75	0.02	0.103	9.87
				CB3	10	9	0,13	9.67	-17.41	0.021	0.061	10.77
				Dune A	54	490	0,89	3.26	-18.88	0.011	0.055	6.62
	2016	Gravel Plain	Fog	C14-1	9	22	1,83	6.52	-19.42	0.012	0.025	6.68
				C14-3	29	233	0,46	1.49	-19.39	0.013	0.133	11.32
				C14-5	48	415	0,46	-53.33	-18.76	0.006	0.034	10.43
			Rain	C14-7	68	570	2,41	4.66	-17.76	0.006	0.054	8.79
				C14-9	85	686	2,41	9.81	-15.22	0.011	0.046	6.14
				C14-11	104	830	2,41	7.85	-15.75	0.011	0.061	6.79
				C14-13	123	932	6,54	7.98	-18.19	0.022	0.1	8.3
				C14-15	138	880	6,54	5.81	-19.77	0.041	0.307	10.72
				C14-17	152	1035	6,54	6.07	-17.32	0.024	0.151	9.09
				C14-19	166	1083	15,47	8.1	-15.86	0.022	0.17	12.08
		Dune	Fog	Dune a	50	392	0,13	1.83	-18.62	0.005	0.016	5.25
				Dune ß	50	406	0,13	2.31	-19.23	0.004	0.014	4.25
				Dune γ	50	406	0,13	5.11	-21.3	0.003	0.007	3.51
			Fog	C14-6.5	62	573	2,41	7.91	-21	0.011	0.145	17.09

Surface Soil (n=53)	2015	Gravel Plain	Rain	C14-7	68	570	2,41	4.96	-15.53	0.018	0.12	9.97
				C14-7.5	72	579	2,41	0.21	-19.65	0.02	0.062	8.67
				C14-8	76	615	2,41	7.91	-20.09	0.009	0.054	10.31
				C14-9	85	686	2,41	13.05	-16.66	0.017	0.128	6.97
				C14-10	94	751	2,41	7.55	-13.77	0.039	0.24	8.35
				C14-11	104	830	2,41	10.63	-16.93	0.023	0.14	7.85
			Fog	C28-1	7	55	1,83	8.92	-23.87	0.011	0.058	7.34
				C28-2	25	211	1,83	1.63	-18.68	0.005	0.04	9.23
				C28-3	44	355	1,83	7.07	-20.11	0.004	0.028	11.98
		Dune	Rain	C28-4	64	467	2,41	7.48	-19	0.008	0.03	10.49
				C28-5	84	704	2,41	10.45	-19.02	0.012	0.081	10.61
				C28-6	103	938	11,28	9.17	-17.1	0.028	0.206	9.83
				Dune 1	136	928	4,75	6.73	-18.31	0.005	0.024	7.74
				Dune 2	136	928	4,75	5.3	-19.18	0.004	0.024	7.01
				Dune 3	139	935	4,75	9.71	-17.11	0.036	0.288	11.6
	2014	Gravel Plain	Fog	C14-1	9	22	1,83	10.55	-19.23	0.02	0.07	6.9
				C14-2	19	118	1,83	8.63	-22.11	0.02	0.19	9.6
				C14-3	29	233	0,46	7.34	-21.79	0.02	0.1	7.51
				C14-4	38	312	0,46	6.46	-19.22	0.01	0.05	8.52
				C14-5	48	415	0,46	13.38	-14.95	0.01	0.05	12.99
				C14-6	58	535	2,41	-18.31	-19.41	0.003	0.02	14.89
			Rain	C14-7	68	570	2,41	-4.78	-16.67	0.014	0.07	7.76
				C14-8	76	615	2,41	9.14	-17.53	0.012	0.06	7.27
				C14-9	85	686	2,41	5.14	-15.16	0.03	0.12	7.7
				C14-10	94	751	2,41	6.14	-13.76	0.03	0.19	8.35
				C14-11	104	830	2,41	9.42	-15.21	0.02	0.17	10.17
				C14-12	113	936	6,54	9.03	-14.74	0.06	0.3	7.51
				C14-13	123	932	6,54	9.67	-16.25	0.04	0.22	8.79
				C14-14	132	881	6,54	9.73	-18.42	0.05	0.37	9.7
				C14-15	138	880	6,54	5.77	-20.44	0.05	0.32	10.77
				C14-16	146	960	6,54	6.63	-17.38	0.03	0.23	10.5
				C14-17	152	1035	6,54	6.01	-17.08	0.02	0.12	10.57
				C14-18	161	1085	15,47	4.97	-17.24	0.03	0.34	12.65
				C14-19	166	1083	15,47	5.87	-15.67	0.04	0.41	11.71
				C14-20	172	1255	15,47	5.61	-18.08	0.04	0.3	10.42
		Dune	Fog	CB1	10	9	0,13	7.86	-20.75	0.011	0.017	4.44
				CB2	10	10	0,13	5.8	-22.71	0.006	0.012	3.63
				CB3	10	9	0,13	7.48	-21.31	0.01	0.014	4.96
				Dune A	54	490	0,89	0.63	-20.95	0.003	0.015	4.27
Subsurface Soil (n=47)	2016	Gravel Plain	Fog	C14-1	9	22	1,83	6.73	-19.46	0.01	0.023	5.78
				C14-5	48	415	0,46	-0.74	-21.75	0.004	0.034	10.41
			Rain	C14-7	68	570	2,41	5.16	-17.43	0.007	0.035	6.79
				C14-9	85	686	2,41	11.61	-16.21	0.009	0.07	7.59
				C14-11	104	830	2,41	4.91	-15.74	0.011	0.054	7.29
				C14-13	123	932	6,54	9.94	-18.46	0.031	0.195	8.21
				C14-15	138	880	6,54	6.51	-18.66	0.041	0.245	9.35
				C14-17	152	1035	6,54	6.17	-15.72	0.017	0.096	9.44
				C14-19	166	1083	15,47	8.33	-15.88	0.021	0.15	11.67
		Dune	Fog	Dune a	50	392	0,13	4.11	-19.03	0.006	0.033	6.67
				Dune ß	50	406	0,13	2.66	-17.2	0.005	0.018	5.04
				Dune γ	50	406	0,13	4.01	-17.53	0.005	0.021	4.94
	2015	Gravel Plain	Fog	C14-6.5	62	573	2,41	6.87	-19.44	0.013	0.046	8.17
			Rain	C14-7	68	570	2,41	5.33	-18.46	0.006	0.048	8.7
				C14-7.5	72	579	2,41	2.73	-18.87	0.012	0.04	10.78
				C14-8	76	615	2,41	18.69	-18.8	0.009	0.062	10.46
				C14-9	85	686	2,41	18.67	-16.74	0.009	0.095	7.99
				C14-10	94	751	2,41	8.08	-15.41	0.029	0.17	8.97
				C14-11	104	830	2,41	11.6	-16.37	0.022	0.13	9.84
			Fog	C28-1	7	55	1,83	9	-20.08	0.015	0.071	8.64
				C28-2	25	211	1,83	2.47	-21.42	0.005	0.04	8.51
				C28-3	44	355	1,83	7.91	-20.96	0.003	0.028	13.78
		Dune	Rain	C28-4	64	467	2,41	7.78	-14.91	0.035	0.064	8.82
				C28-5	84	704	2,41	10.71	-18.6	0.012	0.088	9.38
				C28-6	103	938	11,28	9.25	-14.75	0.019	0.167	10.39
				Dune 1	136	928	4,75	5.93	-17.71	0.007	0.035	6.69
				Dune 2	136	928	4,75	5.54	-17.23	0.007	0.025	6.12
				Dune 3	139	935	4,75	9.47	-16.15	0.026	0.154	10.13
			Fog	C14-1	9	22	1,83	5.71	-19.06	0.01	0.03	11.25
				C14-2	19	118	1,83	7.24	-20.17	0	0.05	11.79
				C14-3	29	233	0,46	7.16	-14.19	0.01	0.11	17.58
				C14-4	38	312	0,46	-0.7	-12.11	0.01	0.07	16.74
				C14-5	48	415	0,46	5.98	-14.91	0.02	0.07	15.65
				C14-6	58	535	2,41	7.25	-18.85	0	0.02	7.74

			C14-7	68	570	2,41	4.54	-28.24	0.01	0.06	7.99
			C14-8	76	615	2,41	3.73	-19.7	0.01	0.04	7.6
			C14-9	85	686	2,41	6.58	-12.44	0.02	0.1	11.22
2014	Gravel Plain		C14-10	94	751	2,41	5.08	-12.27	0.02	0.1	8.63
			C14-11	104	830	2,41	8.37	-15.07	0.02	0.12	8.67
			C14-12	113	936	6,54	9.22	-14.24	0.03	0.17	8.59
		Rain	C14-13	123	932	6,54	14.04	-15.8	0.05	0.24	9.58
			C14-14	132	881	6,54	9.1	-17.13	0.04	0.26	10.8
			C14-15	138	880	6,54	6.29	-18.94	0.03	0.25	10.6
			C14-16	146	960	6,54	7.04	-17.1	0.03	0.25	11.43
			C14-17	152	1035	6,54	5.1	-15.15	0.02	0.09	11.71
			C14-18	161	1085	15,47	6.65	-17.3	0.01	0.1	11.68
			C14-19	166	1083	15,47	6.41	-15.52	0.02	0.24	14.78
			C14-20	172	1255	15,47	5.62	-17.89	0.02	0.2	12.45

REFERENCES

Lancaster, J., Lancaster, N. & Seely, M. K. Climate of the central Namib Desert. *MADOQUA* **14,** 5-61 (1984).

Eckardt, F. D. *et al.* The nature of moisture at Gobabeb, in the central Namib Desert. *J. Arid Environ.* **93,** 7-19 (2013).

Harris, I., Jones, P.D., Osborn, T.J. & Lister, D.H. Updated high-resolution grids of monthly climatic observations - the CRU TS3.10 Dataset. Int. J. Climatol. 34, 623-642 (2014).

Supplementary Table 2. Monthly precipitation measurements of 13 relevant SASSCAL weather stations of the central Namib Desert (Kaspar et al., 2015) for March and April 2014, 2015 and 2015. Numbers in brackets indicate the total number of days when the recorded monthly precipiations exceeded 5mm precipiation. *: day with recorded precipitations over 20mm.

	Weather Station ID	Latitude	Longitude	Distance to coast (km)	Altitude (m)	Precipitation (mm								
						2014			2015			2016		
						March	April	Total	March	April	Total	March	April	Total
East/West Gravel Plain transect	coastal MET	-23,0563	14,6295	20	94		NA		0.7	1.0	1.7	0.6	0.7	1.3
	Kleinberg FN	-22,9893	14,7279	30	184		NA		1.1	0.2	1.3	1.2	0.2	1.4
	Kleinberg	-22,9895	14,7282	30	185	0	1.8	1.8	0.8	0.8	1.6	0.2	0	0.2
	Sophies Hoogte	-23,0068	14,8929	50	334		NA		6.5 (2)	0	6.5	0.6	0.4	1.0
	Marble Koppie	-22,9695	14,9897	50	421		NA		3	0.2	3.2	0.7	1.9	2.6
	Vogelfederberg	-23,098	15,029	60	501		NA		0.6	0	0.6	0.1	0.1	0.2
	Garnet Koppie	-23,1154	15,305	90	733		NA		1.2	0	1.2	11.7 (1)	0	11.7
	Ganab	-23,1218	15,5383	110	1002	22.3 (3*)	6.1 (3)	28.4	1.0	0	1.0	0	0	0
	Rooisand	-23,2945	16,1147	170	1176		NA		16.6 (10)	15.2 (3)	31.8	19.2 (4)	2.6	21.8
North/South Gravel Plain transect	Station 8	-23,2653	15,0563	60	487		NA		0.4	0	0.4	0.6	0.6	1.2
	Aussinanis	-23,4435	15,0459	60	405		NA		0	0	0	0.1	1.5	1.6
	Gobabeb MET	-23,5604	15,041	60	406		NA		0.9	0	0.9	0	0	0
Extreme West Dune	Dieprivier	-24,1296	15,8947	150	1056	62.0 (4**)	1.2	63.2	0.8	28.5 (3*)	29.3	2.7	8.0 (3)	10.7

Kaspar F., Helmschrot J., Mhanda A., Butale M., de Clerq W., Kanyanga J. K., Neto F. O. S., Kruger S., Castro Matsheka M., Muche G., Hillman T., Josenhans K., Posada R., Riede J., Seely M., Ribeiro C., Kenabatho P., Vogt R. and Jürgens N. (2015) The SASSCAL contribution to climate observation, climate data management and data rescue in Southern Africa. Adv. Sci. Res. 12, 171-177.
